# Emergency Management of a Spontaneous Rupture of the Superior Thyroid Artery via Pharyngeal Foley Balloon Tamponade: A Case Report

**DOI:** 10.7759/cureus.97133

**Published:** 2025-11-18

**Authors:** Ghezlan Aldawas, Retaj Alawadhi, Imtiyaz N Bhat, Ahmad Alrasheedi

**Affiliations:** 1 Otolaryngology - Head and Neck Surgery, Farwaniya Hospital, Kuwait City, KWT; 2 Otolaryngology - Head and Neck Surgery, Ministry of Health, Kuwait City, KWT; 3 Medicine and Surgery, Royal College of Surgeons in Ireland, Dublin, IRL

**Keywords:** airway emergency, computed tomography angiography, foley balloon tamponade, hemostasis, multidisciplinary management, oropharyngeal hemorrhage, superior thyroid artery rupture

## Abstract

Spontaneous rupture of the superior thyroid artery (STA) is a very rare cause of oropharyngeal hemorrhage and airway emergency. The majority of cases occur secondary to aneurysm, trauma, post-surgical complications, or iatrogenic causes.

In this report, we present the case of a 27-year-old, previously healthy male who arrived with sudden-onset neck swelling, stridor, and massive oropharyngeal bleeding. On arrival, the airway was immediately secured through suction-assisted endotracheal intubation. Computed tomography angiography (CTA) revealed active extravasation from the STA, without evidence of aneurysm. Hemostasis was achieved by inserting a pharyngeal Foley balloon tamponade as a minimally invasive intervention, avoiding the need for surgical ligation or endovascular embolization. The patient recovered without complications and remained symptom-free at six-month follow-up.

This case highlights the rarity of spontaneous STA rupture and emphasizes the potential effectiveness of pharyngeal Foley balloon tamponade as a minimally invasive technique, which can serve both as an immediate first-line intervention and as definitive management, providing safe and sustained long-term outcomes. This underscores the importance of multidisciplinary collaboration in the diagnosis and management of such challenging cases.

## Introduction

Spontaneous rupture of the superior thyroid artery (STA) is a rare and potentially life-threatening cause of oropharyngeal hemorrhage and airway compromise [1]. Most reported cases occur secondary to trauma, aneurysm formation, or iatrogenic injury, while spontaneous rupture without identifiable pathology remains exceedingly uncommon [2,3].

In this case report, we present a 27-year-old male with spontaneous STA rupture, successfully managed with pharyngeal Foley balloon tamponade, as a minimally invasive and definitive intervention [4].

This case underscores the rarity of spontaneous STA rupture and highlights the potential efficacy of pharyngeal Foley balloon tamponade as a minimally invasive intervention, which can serve not only as an immediate first-line measure but also as definitive management, yielding safe and sustained long-term outcomes. It further emphasizes the critical role of multidisciplinary collaboration in the timely diagnosis and comprehensive management of such complex, challenging cases.

## Case presentation

A 27-year-old, previously healthy male with no history of trauma, prior surgery, anticoagulant use, smoking, alcohol consumption, thyroid disease, hypertension, malignancy, or family history of vascular or endocrine disorders, presented to the Emergency Department with sudden-onset right-sided neck swelling, stridor, and massive oropharyngeal bleeding. The symptoms began less than an hour before arrival.

On arrival at the resuscitation area, the patient was in severe respiratory distress. The oral cavity was filled with blood, obscuring visualization of the airway. The anesthesia and ICU teams performed a difficult, suction-assisted laryngoscopy and endotracheal intubation. During intubation, bleeding became massive, with an estimated blood loss of nearly 2 L, leading to hemorrhagic shock. The massive transfusion protocol (MTP) was activated, and he received uncross-matched packed red blood cells initially, followed by additional blood products in a 1:1 ratio with fresh frozen plasma. His hemoglobin dropped from 14 g/dL at presentation to a nadir of 7 g/dL, and he required norepinephrine infusion to maintain hemodynamic stability. He was transferred to the ICU for further stabilization and management.

**Figure 1:**
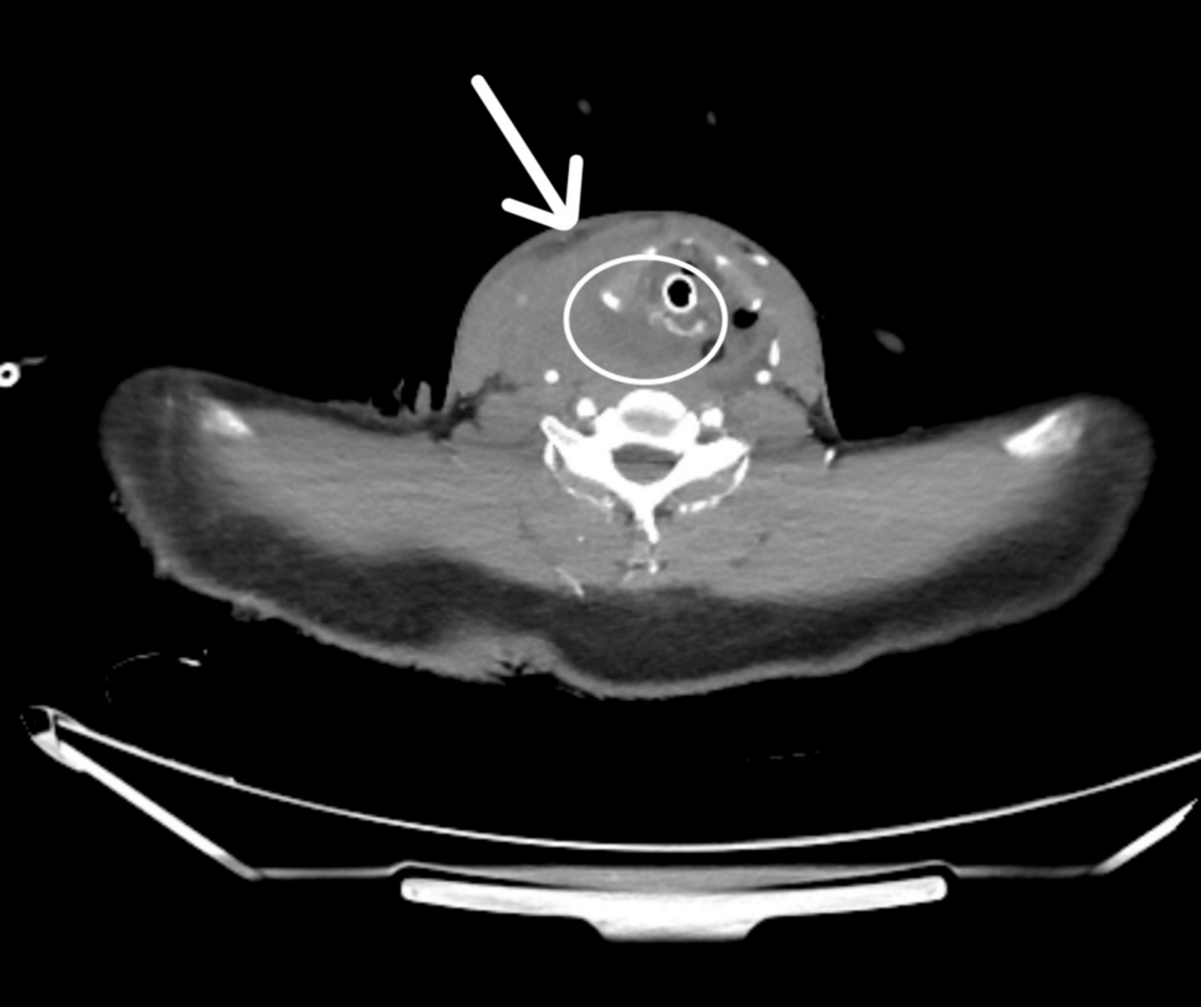
Contrast-enhanced CTA of the neck showing active extravasation from the right superior thyroid artery. CTA demonstrates contrast leak (arrow) from the right superior thyroid artery with surrounding cervical hematoma. CTA, computed tomography angiography

An urgent, contrast-enhanced computed tomography angiography (CTA) of the neck demonstrated contrast extravasation from the right STA, with a large cervical hematoma but no evidence of aneurysm or vascular occlusion (Figure 1). 

**Figure 2:**
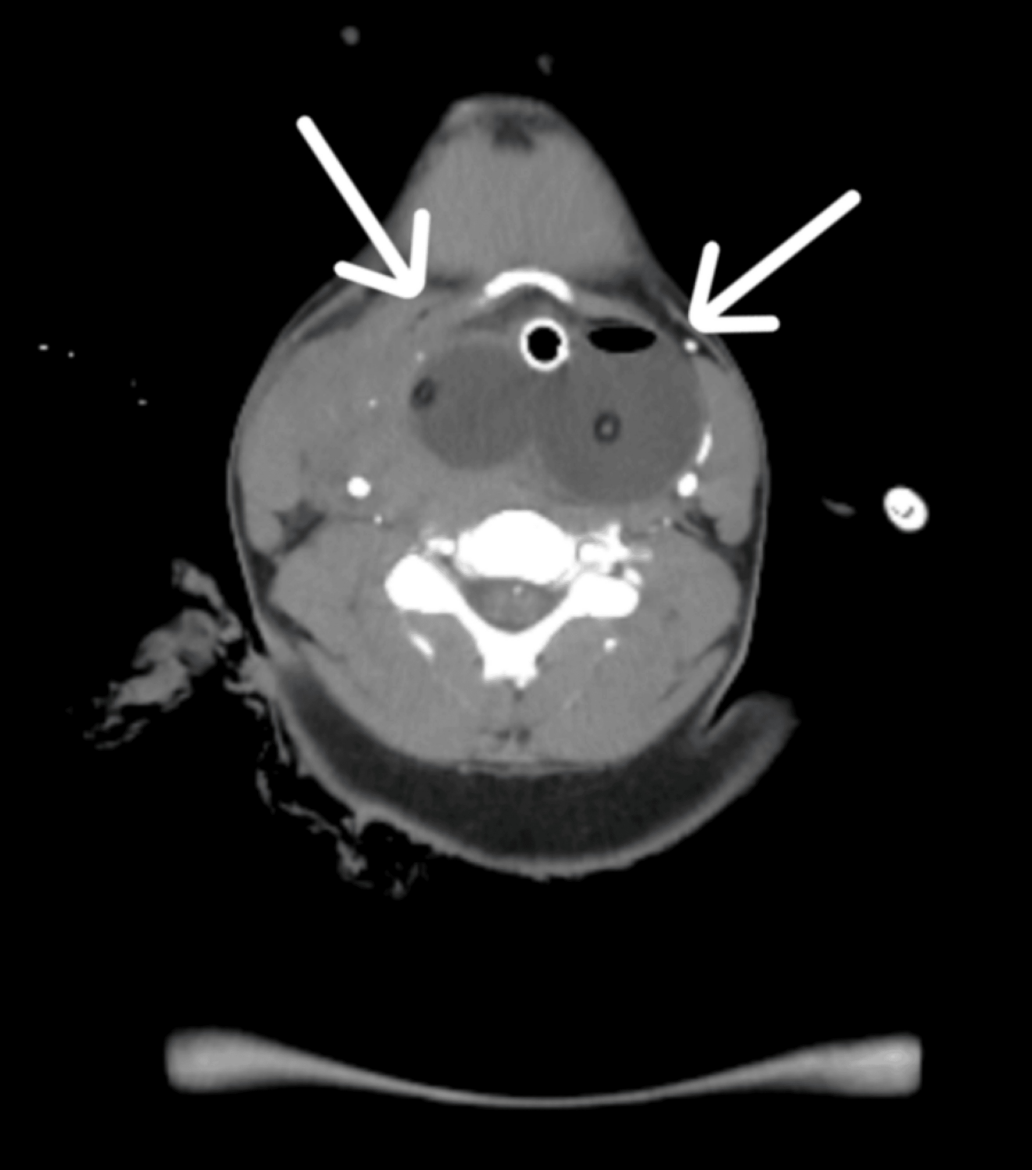
Two pharyngeal Foley balloon catheters in situ at the bleeding site. CT image showing two pharyngeal Foley balloon catheters in situ at the bleeding site (arrows), achieving immediate tamponade and hemostasis. CT, computed tomography

The case was reviewed by a multidisciplinary team comprising ENT, vascular surgery, and interventional radiology. The ENT team took the lead in definitive hemostasis, with two pharyngeal Foley balloon catheters inserted orally and inflated at the bleeding site, achieving immediate control of hemorrhage (Figure 2).

**Figure 3:**
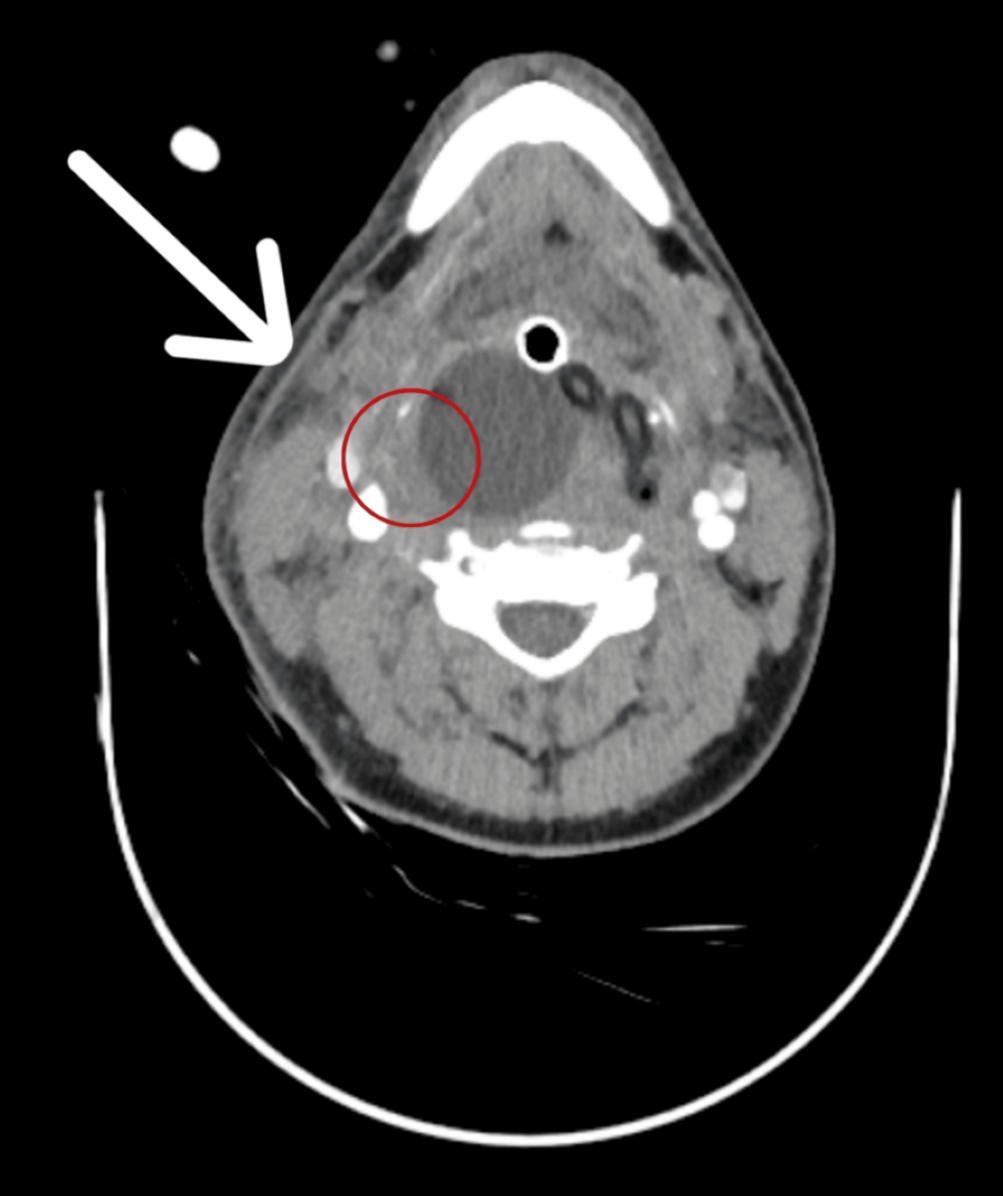
CTA neck after 72 hours. ‏CTA neck after 72 hours showing marked regression of the right-sided hematoma and complete resolution of contrast extravasation (arrow, red circle). CTA, computed tomography angiography

The patient remained intubated on mechanical ventilation, with continuous sedation. A repeat CTA, performed after 72 hours, confirmed resolution of contrast extravasation and regression of the hematoma (Figure 3). Prior to balloon removal, interventional radiology reassessed the patient and confirmed no active bleeding or vascular abnormality. The Foley balloon was deflated and removed five days later, under controlled conditions in the operating theater, with no evidence of rebleeding or complications.

Subsequently, he was weaned off sedation and extubated after successfully passing a spontaneous breathing trial. He maintained stable oxygen saturation on a nasal cannula, tolerated oral intake without difficulty, and gradually resumed mobilization with physiotherapy. He was discharged from the ICU to the surgical ward in stable condition.

Follow-up in the ENT outpatient clinic continued for six months. The patient remained symptom-free, with no recurrence of bleeding, stridor, dysphagia, or dysphonia. Examination of the neck and airway was normal; chest imaging was unremarkable, and a repeat CTA at six months confirmed resolution with no recurrence.

## Discussion

Spontaneous rupture of the STA is an uncommon but potentially life-threatening cause of oropharyngeal bleeding and airway compromise [1,2]. The STA arises from the external carotid artery, characteristically as its first branch. It ensures the supply of blood to the thyroid gland, laryngeal structures, and adjoining muscles [1,3]. The spread of bleeding toward the oropharynx can be explained by the anatomical continuity of the deep cervical fascial spaces, mainly the pretracheal and retropharyngeal planes, which communicate with the upper airway. Through these loose connective tissue planes, extravasated blood from the STA may breach the inferior pharyngeal mucosal layer and extend superiorly into the oropharynx. Minor vascular anastomoses between the superior thyroid and superior laryngeal arteries may further facilitate this spread, accounting for the observed oropharyngeal involvement [1,3,5]. Therefore, given the close anatomical proximity of this artery to the laryngeal inlet, hemorrhage can rapidly propagate into the upper airway, potentially resulting in neck swelling, stridor, and significant oropharyngeal bleeding, which may lead to acute airway obstruction.

Airway management in such cases is difficult due to blood obscuring the field. Decision-making in these situations requires early involvement of a multidisciplinary team, including ENT, vascular surgery, and interventional radiology, to ensure airway protection and definitive hemostasis. Surgical exploration, which is most commonly used in cases of STA bleeding, poses the risk of disrupting the laryngeal structures and surrounding anatomy [5,6]. However, in our case, pharyngeal Foley balloon tamponade was successful as a definitive option for hemostasis, using an ENT-accessible technique without the need for interventional radiology, embolization, or surgical ligation.

The pathophysiology of STA rupture differs depending on the cause. Most cases are iatrogenic, usually after thyroidectomy or radical neck dissection. Trauma, blunt or penetrating, is another common etiology [5,6]. Less frequent causes include aneurysm formation, arteritis, or malignant invasion of the artery [7,8]. Rupture from an aneurysm occurs due to focal vascular wall weakness and degeneration [7,8].

Beyond iatrogenic and traumatic causes, several pathophysiologic processes may contribute to arterial wall fragility, including degenerative changes of the medial layer, immune-mediated vasculitides, and acute hemodynamic surges (e.g., hypertensive spikes or Valsalva maneuvers). Endocrine hypermetabolic states, such as thyrotoxicosis, can further exacerbate vascular wall stress through increased cardiac output and hyperdynamic circulation [9-12].

However, in this rare case presentation, the patient’s artery ruptured spontaneously. There is no history or medical evidence of aneurysm, traumatic injury, or iatrogenic insult. Such a case of spontaneous bleeding is very rare, with only a few instances mentioned in the prior literature [13,14]. It is postulated that the patient might have had weakness of the arterial wall due to subclinical inflammatory changes, unnoticeable trauma while moving the neck, or a spontaneous rise in intracranial pressure while coughing [13]. While reviewing the relevant literature, it was found that Jims et al. [14] and Hanashiro et al. [7] presented similar cases leading to airway obstruction. The case required prompt control of the airway and a subsequent vascular procedure. Jang and Kim [15] presented a case of endovascular management of thyroid artery bleeding, finding embolization to be highly effective, with low complication rates, particularly for aneurysmal or iatrogenic causes. However, the majority of reported patients underwent invasive procedures, either open surgical ligation or endovascular embolization, shortly after diagnosis. Such rare presentations make treatment decisions challenging. The primary priority in such presentations remains airway protection. Rapid progression to airway compromise due to hematoma expansion or massive hemorrhage necessitates early, definitive airway management. In our patient, suction-assisted laryngoscopy allowed intubation despite massive bleeding, preventing hypoxic injury and facilitating further resuscitative measures.

Once the airway is secured, diagnostic imaging is critical. Urgent CTA is the modality of choice for localizing the bleeding source, detecting active extravasation, identifying aneurysms, and guiding intervention. In our case, CTA confirmed STA extravasation without aneurysm or other vascular lesions, supporting consideration of conservative measures once the bleeding was controlled. Repeat CTA after 72 hours demonstrated resolution, confirming the stability of nonoperative management.

In our patient, balloon tamponade was chosen as an initial and ultimately definitive measure. However, in selected patients, such as ours, balloon tamponade may obviate the need for invasive procedures, provided strict monitoring and repeat imaging confirm hemostasis. The risks and benefits of this approach were carefully weighed. Potential complications of conservative management include rebleeding, airway obstruction from balloon migration, mucosal injury, and missed vascular pathology. On the other hand, not performing a surgical procedure or embolization immediately lowers the risk of vascular insult, neurological complications, and other surgical complications. In this case, cessation of bleeding was achieved immediately and sustained, with rapid improvement of vital signs. Clinical decision-making for avoiding surgery was supported by a multidisciplinary approach, in line with the SCARE (Surgical Case Report) guidelines [16].

Our patient’s favorable outcome - with survival and no recurrence at six months - supports the judicious use of conservative management in rare, carefully selected cases of STA rupture. While embolization or surgical ligation remains the standard in most reports, this case demonstrates that promptly controlling the airway, ensuring accurate diagnosis, maintaining hemostasis, and using minimally invasive measures can provide safe and effective management.

## Conclusions

Spontaneous rupture of the STA is an extremely rare but potentially life-threatening cause of oropharyngeal hemorrhage and airway compromise. Most reported cases have been related to trauma, aneurysm formation, or iatrogenic injury. Management has traditionally relied on surgical ligation or endovascular embolization; however, both approaches carry inherent risks and require specialized expertise. Our case demonstrates that minimally invasive pharyngeal Foley balloon tamponade can achieve immediate hemostasis and serve as a definitive management option.

This case emphasizes the importance of considering STA rupture in the differential diagnosis of patients presenting with acute neck swelling, stridor, and oropharyngeal bleeding, even in the absence of trauma or prior surgery. Prompt airway protection, urgent imaging with CTA, and a multidisciplinary approach are essential for favorable outcomes. Early recognition and appropriate treatment are crucial to prevent complications and improve patient outcomes. Continued research and clinical collaboration are essential to enhance understanding and management of this complex condition.
